# Editing cholesterol out of the blood

**DOI:** 10.1038/s43856-021-00011-5

**Published:** 2021-06-30

**Authors:** Ben Abbott

**Affiliations:** Communications Medicine, http://www.nature.com/commsmed

## Abstract

In patients with high cholesterol and at risk of cardiovascular disease, inhibitors of PCSK9 are useful in lowering lipid levels but must be dosed regularly. A recent study in *Nature* by Munsunuru and colleagues explores the possibility of permanently disrupting *PCSK9* expression via in vivo CRISPR gene editing in non-human primates, with long-lasting reductions in LDL cholesterol.

**Figure:**
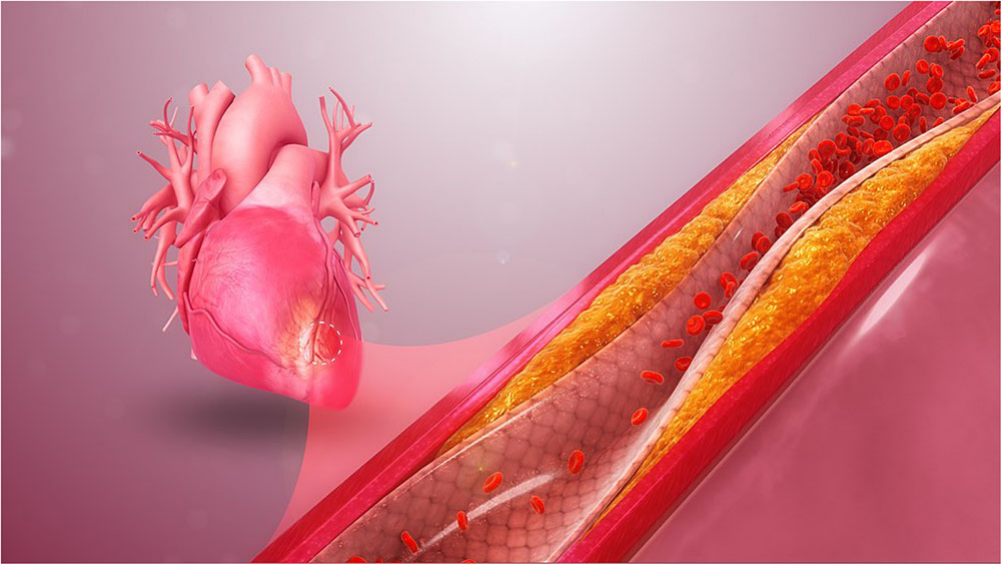
https://www.scientificanimations.com, CC BY-SA 4.0 https://creativecommons.org/licenses/by-sa/4.0, via Wikimedia Commons

High LDL cholesterol is a causal risk factor for atherosclerotic cardiovascular disease, the leading cause of deaths globally. The liver protein PCSK9 regulates the level of circulating LDL and is an established target for lipid-lowering therapies, including monoclonal antibodies and small interfering RNAs. *PCSK9*-targeted antisense oligonucleotides are also being tested in animal and human studies. However, all of these therapies require routine dosing to maintain lower cholesterol levels, with the risk of patient non-adherence to treatment.

Therapeutic gene editing—which holds enormous potential for a number of clinical indications—might provide a solution to this problem: a strategy to permanently reduce *PCSK9* expression. In vivo CRISPR base editing enables the precise and stable modification of single nucleotides in the genome, correcting causative mutations or inactivating disease genes. To this end, Munsunuru and colleagues evaluated the feasibility, safety and efficacy of targeting wild-type *PCSK9* using a CRISPR adenine base editor in cells grown in vitro and in living mice and non-human primates^1^.

The authors formulated lipid nanoparticles containing the ABE8.8 adenine base editor, which can replace certain adenine residues with guanine, combined with guide RNAs to direct the base editor to a specific splice site within *PCSK9*. After first confirming disruption of this site in primary human hepatocytes in vitro, they administered a range of doses to mice intravenously and achieved an editing efficiency of 70% within the liver, the main site of *PCSK9* expression in the body.

Editing efficiency was similar in the livers of cynomolgus monkeys two weeks after dosing with the nanoparticles and was accompanied by an 81% reduction in the level of PCSK9 in the blood and a 65% decrease in LDL cholesterol. In a longer-term study, which is still ongoing, the authors have achieved a stable 90% reduction in circulating PCSK9 and a 60% reduction in LDL up to 8 months post-dosing, which matches or surpasses the effect of current therapies used in patients.

Markers of liver damage were transiently increased by the treatment, although this was determined to be a response to the nanoparticles themselves rather than *PCSK9* editing, and no adverse health effects were observed. Sequencing studies found that off-target editing was minimal.

These findings represent the first efficient delivery of a CRISPR base editor to non-human primates and indicate that in vivo base editing is a precise and effective strategy to target *PCSK9* and durably reduce cholesterol levels. Although further evaluation of the safety and risks of in vivo gene editing in humans will be required, these data represent an important step towards translation of *PCSK9* editing into the clinic, whereby a ‘once-and-done’ approach would bring significant benefits for patients.
